# Multisport-Integrated Training for Rugby Instructors: Success and Effects on Minirugby Players

**DOI:** 10.3390/jfmk10010011

**Published:** 2024-12-27

**Authors:** Marta Rigon, Gabriele Signorini, Raffaele Scurati, Athos Trecroci, Dario Colella, Damiano Formenti, Giampiero Merati, Domenico Cherubini, Pietro Luigi Invernizzi

**Affiliations:** 1 Department of Biomedical Sciences for Health, Università degli Studi di Milano, 20133 Milan, Italy; marta.rigon@unimi.it (M.R.); gabriele.signorini@unimi.it (G.S.); athos.trecroci@unimi.it (A.T.); pietro.invernizzi1@unimi.it (P.L.I.); 2 Sport Faculty, San Antonio Catholic University of Murcia, 30107 Murcia, Spain; dcherubini@ucam.edu; 3 Department of Biological and Environmental Sciences and Technologies, University of Salento, 73100 Lecce, Italy; dario.colella@unisalento.it; 4 Department of Biotechnology and Life Sciences, Università degli Studi dell’Insubria, 21100 Varese, Italy; damiano.formenti@uninsubria.it (D.F.); giampiero.merati@uninsubria.it (G.M.); 5 IRCCS Fondazione don Carlo Gnocchi, 20148 Milan, Italy

**Keywords:** teaching styles, teaching competence, system thinking, motor competence, sports education

## Abstract

Background/Objectives: The educational system thinking approach (ST) takes a holistic vision of instructors/teachers and learners’ relationships, making sports pivotal for reflection on education. This study evaluated the efficacy of a multisport ST-based course on minirugby instructors’ teaching competence and children players’ motor conduct. Methods: The twenty-five rugby instructors (IAC) attended the 25 h course and the children of their teams (n = 109, Ch-IAC) participated in this study as experimental groups. Twenty-five rugby instructors who were not attending the course (I-CON) and their pupils (n = 111, Ch-CON) acted as control groups. Changes in instructors’ teaching competence (by the Instrument for Identifying the Teaching Style and the System for Observing Fitness Instruction Time) and children’s motor conduct (by the Körperkoordinationtest für Kinder test, the Game Performance Assessment Instrument, the Physical Activity Enjoyment Scale, and the physical self-efficacy scale for children) were assessed. Results: Thanks to the education received, IAC improved in didactics and methodological competence. They learned to use more production teaching styles than CON (10.5 ± 9.3% vs. 0% of the lesson time, *p* < 0.05), reduce children’s inactive lesson time for management (−5.1 ± 3.3% vs. 1.1 ± 3.1%, *p* < 0.05) and promote more outside lesson topics (4.4 ± 3.2% vs. 0%, *p* < 0.05). In addition, compared to Ch-CON, Ch-IAC significantly improved motor coordination, game performance, enjoyment, and self-efficacy (*p* < 0.05). Conclusions: Children’s enjoyment and self-efficacy acted as mediators that amplified the effects of the multisport training course. At the same time, the instructor’s didactical and methodological competence were moderators directly favoring or worsening children’s motor competence. Such an integrated multisport model is applicable and suggested for improving sports performance and education processes.

## 1. Introduction

Sport can be assumed as a complex organism composed of a broader “systemic constellation” of parts that cannot be reductively approached by a narrow and independent analysis of the separate elements that compose it. In this complexity of interconnections, the educational system thinking approach (ST) takes a holistic vision of the roles of instructors/teachers and learners in their own reality [1].

The term *educational system* refers to a complex web of related elements interacting with each other and representing a coherent, unique entity of youth sports. Examples of integrated and interconnected elements leading to sport and education success are (i) the reference context and the training vision; (ii) the reference legislation; (iii) the administrative sphere; (iv) the technical, pedagogical, personal, and relational skills of the instructors; (v) the children’s motor and psycho-social skills.

In minirugby (the youth sector of rugby where children aged 5 to 12 learn the basics of rugby through simplified rules, reduced impacts, smaller field dimensions, and fewer players), the setting, the activities, and the social dynamics are key elements that can enhance the holistic approach based on the ST model. Each element can be structured over levels, from proximal to distal [2,3,4], such as follows:

- Setting: The methodological organization/structure of the minirugby program, the structure of the rugby club, and the broader structure of the community in which the club is embedded.
- Activities: Practicing the sport of interest, considering other physical/sports activities in different contexts, and engaging in complementary socio-cultural activities (school, art, music).
- Social dynamics: Within the specific sports context (instructor-child, child-child, and parent-child relationships), within complementary sports activities and group dynamics (interdependence, cohesion, identity), and within the socio-cultural sphere (external relationships with public and private entities).

A similar holistic approach was already applied in a previous study on minirugby to assess athletes and instructors’ skills, didactics and relationships [5]. The ST approach must translate into positive outcomes over time in the short (having the child fun and being engaged), medium (specific skills and connections acquired should be assessed), and long term (expecting success in sports, academics, and life), hopefully guaranteeing the child’s complete personal development.

Therefore, academic educational research must be precisely oriented to the concrete needs of practice and the specifics of the contexts it addresses to be truly useful in addressing the complex problems of youth sports education. This is crucial for developing effective educational policies and practices in youth sports [6]. The social reporting concept [7] implies that intervention is sustainable and justified by tangible positive results, which, in sports, are generally accounted for through successful performances, but not limited to that since recently shifted towards the educational value of sport [8], which strengthened reflection on education’s methods, objectives, and outcomes.

The broader educational role of sport is particularly facilitated by capable instructors who know how to stimulate the child towards varied motor experiences to capture their attention and interest in addition to the proposal of usual specific discipline skills. This way, teaching styles are recognized as fundamental methodological elements in an instructor’s professional competence [9]. Therefore, the training of sports instructors must include a strong focus on didactic-methodological skills, which are essential in educational processes and beneficial beyond sports and life in general. It is important to note that participation in a sporting activity alone does not always generate benefits [10,11,12].

Effective educational communication is crucial: it supports people’s successful involvement in sports and physical activity. In the athlete–coach relationship, communication is the key that unlocks the development of knowledge, understanding, and sharing of goals and beliefs, reassuring both parties and building a strong foundation for success [13]. One type of supportive communication is based on the Self-Determination Theory, founded on the three following needs necessary for educational success: (i) autonomy (i.e., the possibility to act as “actors” in one’s actions); (ii) competence (i.e., feeling effective and confident in one’s abilities, skills, and actions); (iii) relationship (i.e., feeling a sense of meaningful and reciprocal connection with others) [14]. Proper instructors’ verbal and non-verbal language, voice and paralanguage, specific organizational capacity, and psychological and personal competence represent the basics of effective and need supportive communication [14,15,16].

Multisport practice, as a multi-teaching approach, and improvement in instructors’ teaching skills (involving need-supportive communication skills) can represent a concrete operational translation, which, when applied, significantly enhances young people’s motivation to participate in sports. Through this integrated approach, it is possible to manipulate information and instruction about the needs of young people, thereby helping them to develop and manage their motivation for sports participation [17]. Multisport practice allows children to learn to choose autonomously and to develop various proposals practical for creating elastic motor maps that can be translated into the ability to adapt/transform the motor competence to the required needs of different situations and contexts [18].

An integrated approach, composed of multisport, multi-teaching, didactic, and organizational competence, makes it possible to orient teaching in a multi-purpose manner and target more towards the physical-motor, cognitive, or psycho-social areas, allowing flexibility of intervention adapted to the specific realities and educational needs of young people. The integrated approach plays a crucial role in managing educational interventions effectively. It ensures that teaching is targeted and efficient by minimizing downtime and periods of inactivity [19,20].

A scientific-based verification study of a methodological process of the training intervention is necessary to propose an integrated model that warrants complete didactic transferability in youth sport instructor courses [21]. Specifically, the present model, based on that by Kirkpatrick and Kirkpatrick [22], is characterized by the following phases:

1. Theoretical reference training (in this case, model-integrated and multisport information).
2. Proactive workshop formula, focusing on sharing, deciding and formulating teaching objectives, discussing these with the learners and reflecting on didactic-methodological decisions.
3. Organization of traineeships (congruent with the knowledge of the theoretical topics covered) with proposals to be formulated by teachers for their colleagues and to children from different sports categories.
4. Analysis of the effects of the training proposal on teachers’ teaching ability and of the transference that these effects present on the motor competence, enjoyment, perception of self-efficacy; amount of physical-motor commitment of the children attending the courses in which these instructors carry out their educational activities.
5. Evaluation of instructors’ satisfaction with the training course.
6. Comparison of the effects of this training with those of youth sports instructors who follow the sports federations’ standard, predominantly sport-specific and technical training.

This study aimed to evaluate and compare the effects of a multisport-integrated model training, oriented to emphasize the didactics and methodological competencies of the rugby instructors and, indirectly, on the effects (educational and sports success) of the training course on motor competence, game performance, self-perception and enjoyment in minirugby players. More specifically, this study investigated how a typology of integrated, multilateral, multi-purpose, and multisport training models for instructors can determine a direct transferability of results in terms of *body education* and *education through the body* in children attending specific sport introduction courses. We hypothesized that this training process significantly improves teachers’ teaching ability and awareness about the effects of teaching by models-integrated/multisport methodology and the teaching styles they use. It is expected that the improvement of the didactic-methodological capacity translates into evident results on a psychomotor and psychological level in the training of the youth teams entrusted to the instructors benefiting from the specific multisport education compared to those who follow a traditional single-sport training mainly of technical nature.

## 2. Materials and Methods

### 2.1. Study Design

In this study, rugby instructors were recruited to evaluate the outcomes of the multisport-integrated training program, and the children belonging to each instructor’s team were involved in estimating possible changes in motor and psycho-social competencies resulting from the instructors’ training process. The sample size (n = 45) was previously calculated with the G*Power (ver. 3.1.9.2) program using a statistical power of 95% and an effect size estimate (Cohen’s d) of 0.5. For this calculation, the student *t*-test for unpaired data was chosen. Since 25 rugby instructors joined the multisport-integrated training program, 25 FIR rugby instructors from another rugby club were required. The statistical power resulting from this instructor sample was 0.81. Therefore, a minimum of 210 total pupils was necessary to satisfy the previously set statistical power.

### 2.2. Participants

A total of 50 FIR rugby instructors from teams of the Milan area and 220 children of U10 and U12 categories (the children who played in the rugby instructors’ team) participated in this study (Table 1). To be included in this study, instructors had to have an FIR training license and a minimum of two years of training experience but no bachelor’s degrees in sports sciences. The instructors of the experimental group must have attended at least 75% of the training program lessons. Inclusion criteria for children were attendance at a rugby course and the absence of physical (e.g., muscle-skeletal) and cognitive pathologies (e.g., cognitive disabilities) that could alter the normal procedures of tests. They also attended not less than 75% of training sessions during the experimental period.

The instructors who participated in the training course and the children belonging to their respective teams composed the experimental groups (IAC = Instructors attending the course and Ch-IAC = children of the instructors attending the course); those who did not attend the training course and the respective children acted as control groups (I-CON, and Ch-CON, Instructors- and Children-Control, respectively). I-CON has not received further training in addition to that by federal specialistic rugby (i.e., the inclusion criterium).

Concerning children participating in this study, since women’s rugby in Italy is a developing movement and girls usually approach this sport at a late age (after 12–14 years), the number of female minirugby players is minimal and does not represent a source of data bias. In addition, all children were at prepubertal age. Therefore, we decided not to exclude female players from this study.

### 2.3. Procedure

This study followed the randomized control trial model.

First, the baseline condition (pre) of instructors and children of the experimental and control groups was assessed. The instructors’ initial levels of didactical and methodological competencies were determined: self-reported teaching styles and didactic interventions on their children’s teams were collected, and the teaching approach while leading practice and the resulting amount of physical activity of their children were observed. In addition, motor competence, enjoyment, self-efficacy, physical activity, and play performance of the children belonging to the instructors’ teams were also measured.

Over 6 months, the instructors of the experimental group (IAC) attended a multisport-integrated training and were allowed to immediately apply the newly acquired knowledge during the rugby training sessions with their respective teams (Ch-IAC). The instructors of the control group (I-CON) continued their usual training with the respective children’s teams (Ch-CON). All the children were involved in a training period of 100 h per course distributed in 3 training sessions of approximately six hours per week. Therefore, in Ch-IAC, one session per week was dedicated to multisport activities and two sessions to rugby-specific activities. All three sessions of the Ch-CON were only dedicated to rugby-specific activities.

Measurements were repeated at the end of the multisport training course (post) to evaluate the outcomes on experimental instructors and respective children compared to controls, and further information about IAC satisfaction with the training attended was surveyed.

This study was approved by the ethical committee of the University of Milan.

### 2.4. The Multisport Instructor Training Course for IAC

The multisport instructor training course for IAC was structured as follows:

- Four meetings of 6 h and 15 min per meeting (25 h total—1 University Training Credit);
- A theoretical part in the classroom (2 h 15 min) structured with the following criterion: theory integrated with the subsequent practice of the internship (30/40 min); group work with discussions on the theoretical topics covered (15/20 min); questions from group work (a person in charge collects the requests for clarification that emerged from the previous discussion and addresses them to the trainers, 10 min); preparatory theory for the development of practical lessons (30/40 min); group work aimed at preparing internship interventions (themed lessons and methodological organization of the teaching moment, 15/20 min); questions from group work requesting further clarification on the internship (10 min);
- A practical part on the field, training with the coaches with an alternation in the roles of instructor, student, and observer (2 h);
- A practical part on the field, training with children with instructors who alternate in leading the lessons and observing (2 h).

The training process followed the main directions of the literature in its design [23,24] and was characterized by:

- Proposal to rugby instructors with previous technical-sporting experience on which to base further knowledge and teaching-training strategies, in particular through multisport activities (basketball, ultimate, mini-volleyball, peteca, judo) valid for a specific transfer into the discipline;
- Sufficient duration of the experimentation (six months) and support to the instructors by the trainers, in the classroom and the field, to guarantee the learning and consolidation of the new skills;
- Composition of the team of trainers (scientific coordinator, teaching coordinator, researchers, doctoral students, and graduate students specially trained in administering questionnaires, motor tests, video recording, and analysis).

It was hypothesized that the model used to verify the effectiveness of the proposed educational teaching process satisfies the four key levels for the evaluation of teaching processes [22]:

1. Satisfaction of the instructors with the program received, highlighting strengths and weaknesses;
2. Learning of knowledge, with greater awareness of the educational aspects of the multisport, of the teaching styles that can be used and their effects, of the languages that can be used in communication on the field and the gym through the acquisition of an expressive, empathetic and engaging them;
3. Modification of teaching behavior during internship experiences with coaches and children (ability to use different teaching styles and more effective educational communication; improvement of one’s teaching-organizational skills);
4. Final results achieved (benefits acquired by the children who follow the multisport rugby introductory courses and determined by the educational training process followed by the instructors).

**Figure 1:**
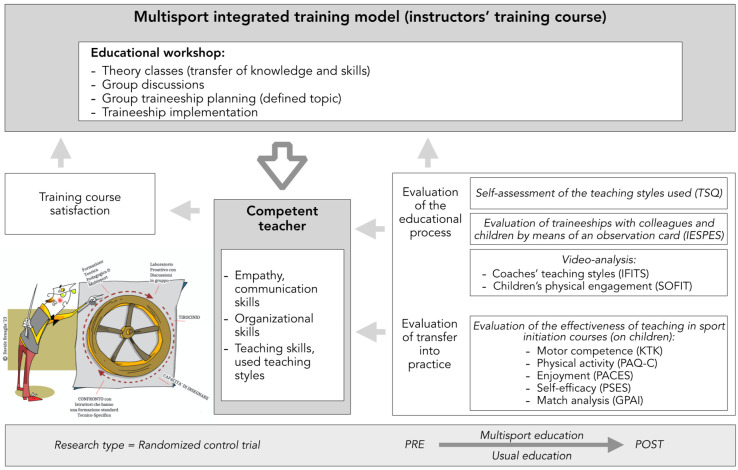
Framework for the methodological process of the training intervention and the investigation using a scientific approach to verify the outcomes [25].

**Figure 2:**
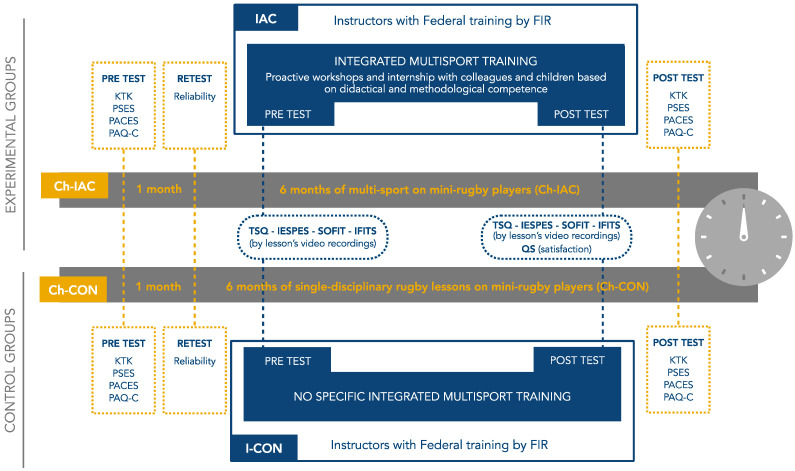
Timeline of the procedures followed by experimental (IAC and Ch-IAC) and control groups (I-CON and Ch-CON).

The multisport-integrated model of the training course and the timeline of procedures for experimental and control groups are shown in Figure 1 and Figure 2.

### 2.5. Measures

The instructors’ methodological and didactic competence were assessed by the following instruments:

- Internship evaluation sheet in physical education and sports (IESPES) modified by Navickiene [26]: It is a tool designed with the aim of measuring the different didactic competencies acquired during training with real practice in the field highlighted in the application context of the internship on children or colleagues involved in the training [22,27]. A score from one to five points is attributed to communication capacity, didactics organization, and capacity of motivation and to engage the pupils [28]. The descriptors of the modified IESPES are detailed in the Appendix A;
- The teaching styles questionnaire (TSQ) [29,30]: It is designed to assess the instructors’ perception of their teaching styles. It is composed of eleven items based on Mosston and Ashworth’s spectrum of teaching styles scored by a 5-point Likert scale corresponding to the frequency of each style occurrence [31];
- The questionnaire of satisfaction (QS) by Kirkpatrick and Kirkpatrick [22]: It is a tool designed to measure the level of satisfaction of participants in the training course. The training program is considered satisfactory if the participating instructors are happy with it and motivated to learn. Participants’ interest, attention and motivation to attend the course are indicators of the program’s appreciation. The questionnaire is scored by a four-point Likert scale and includes thirteen questions. The questionnaire showed a good internal consistency equal to a Cronbach alpha value of 0.836.

Finally, some lessons were video recorded, and the Instrument for Identifying the Teaching Style (IFITS) [32] and the System for Observing Fitness Instruction Time (SOFIT) [33] were used to verify the instructors’ teaching styles and the corresponding children’s levels of physical commitment during the lessons. Two lessons were recorded both before and after the intervention. The IFITS video analysis evaluates the percentage of time spent by the instructors in each teaching style. The SOFIT video analysis evaluates the percentage of time spent by children in statical, moderate to vigorous physical activity. The content of the lessons and the type of the instructors’ interaction were further analyzed.

To further check the success of the multi-teaching training program and possible outcomes differentiating instructors attending the course from those acting as a control, some parameters of children composing the teams led by the instructors were analyzed. Specifically, children’s level of coordination, amount of physical activity, game performance enjoyment, and perception of motor competence were assessed by the following instruments:

- Körperkoordinationtest für Kinder (KTK) [34] is a product test that measures coordination skills. The KTK consists of four items; a final score can be obtained for each test. The scores are added and converted with the reference nomograms by age and gender, returning a motor quotient indicating the level of body mastery possessed by the subject.
- The Physical Activity Questionnaire for Older Children (PAQ-C) [35] aims to determine the level of physical activity in the last 7 days, including sports, recreational activities, dancing, climbing, cycling, and unstructured activities. Low scores (from 1 to 2.33) correspond to a low level, medium scores indicate a moderate level (from 2.34 to 3.66), and high scores (from 3.67 to 5.00) imply a high level of physical activity;
- The game performance assessment instrument (GPAI) [36] aims to evaluate game performance behaviors that demonstrate tactical understanding and the player’s ability to solve tactical problems by selecting and applying appropriate skills. Our study used it as the overall game performance of the whole team. To the different variables of the game (decision making, skills execution, support, game performance), judgments of appropriate/inappropriate and efficient/inefficient responses were given using percentages of total responses. A score is attributed based on an overall division between the number of appropriate versus inappropriate responses, and a score over one indicates more positive than non-positive responses. The GPAI video analyses were performed before and after the intervention.
- The Physical Activity Enjoyment Scale (PACES) [37] is a 16-item questionnaire validated for elementary school students from 12 to 16 years old. PACES scores the items answers based on a 5-point Likert scale (1: completely disagree, 2: disagree, 3: uncertain, 4: agree, 5: fully agree);
- The physical self-efficacy scale for children (PSES) [38] evaluates one’s self-perception of one’s physical efficiency in motor skills. This is considered a primary motivation for voluntary physical activity and sports participation. The test is scored 1–24. The higher the score, the higher the self-efficacy.

A team of Sports Science experts and doctoral students conducted all measurements during the mini-rugby course practice. The entire data collection and intervention took place over eight months. Test reliability and intra/inter-operator reliabilities were performed to ensure the validity of the analysis.

### 2.6. Statistical Analysis

The normality of data distribution was verified using the Shapiro–Wilk test.

The Anova 2 × 2 (time × group) analysis was conducted with the aim of investigating the intervention’s efficacy for IESPES, KTK, and PACES. This involved examining the time effect (pre vs. post), the group effect (experimental vs. control), and the interaction (time × group) for each analysis. The intervention’s efficacy for self-reported teaching styles, IFITS, SOFIT, and PSES, was investigated using delta values (post–pre) and the Mann–Whitney U test (a non-parametric version of the unpaired *t*-test) due to the violation of the normal distribution of data. For all mentioned analyses, the level of significance was set at 0.05.

As the GPAI protocol required the analysis of only four matches (two pre- and two post-intervention), the GPAI data were analyzed using a percentage comparison. In addition, a multiple regression analysis was performed to obtain information about mediators and moderators. The mediation analysis was performed following Baron and Kenny’s method and was aimed at establishing mediation criteria. We considered the intervention (multisport training course) as the independent variable (IV), the main outcome (the motor competence ΔMQ norm, i.e., the delta of normalized motor quotient) as the dependent variable (DV), and the mediators (enjoyment, self-efficacy, and PAQ-C) as the variables controlling the IV-DV relationships. The mediation analysis was performed to evaluate a comprehensive measure of the effect of IV on DV and pre-post changes in the intervention group. A dummy variable was computed to differentiate the two intervention groups: 1 for the experimental group and 0 for the control group. Finally, the moderation analysis was performed to evaluate the role of a multi-teaching approach and the didactics competence of the instructors (as ModV) on children’s motor competence (DV) and if they influenced the multisport training course (IV). A dummy variable of 1 was assigned for the experimental group and 0 for the control group. Concerning instructors’ competence, a value of +1 was attributed to instructors’ higher competence, 0 to instructors’ average competence, and −1 for instructors’ poor competence.

## 3. Results

Data describing the didactic approach of the instructors during teaching as surveyed by the IESPES (Table 2) revealed significant interactions in communication (F = 15.837, *p* < 0.001, ηp^2^ = 0.142), didactics organization (F = 4.63, *p* < 0.001, ηp^2^ = 0.046), motivation/personal competence (F = 18.157, *p* < 0.001, ηp^2^ = 0.159), and the total score (F = 33.896, *p* < 0.001, ηp^2^ = 0.261). Only IAC improved their scores in the post-intervention measurements.

The analysis of the instructors’ self-reported teaching styles surveyed by the TSQ (Table 3) showed that before the training course, IAC reported having performed more multi-teaching (*p* = 0.048) and reproduction (*p* = 0.016) styles occurrences than I-CON during activity conduction. After the intervention, multi-teaching (*p* < 0.001), reproduction (*p* < 0.001), and production (*p* < 0.001) teaching styles of IAC were further different than I-CON. The deltas (post–pre) analysis between the number of reported style occurrences further showed that IAC improved their abilities and taught with a larger number of styles, differently than I-CON, who basically unchanged their teaching approach.

**Figure 3:**
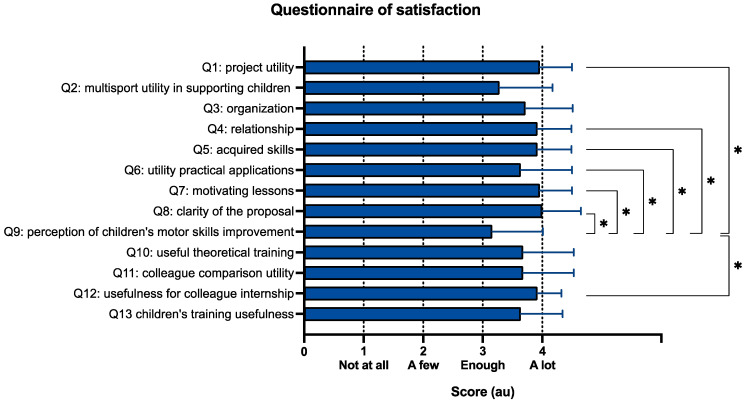
Results of the questionnaire of satisfaction (QS). In the survey, scores from 1 to 4 were assigned based on the level of satisfaction (1—not at all, 2—a few, 3—enough, 4—a lot). Significant difference: * = *p* < 0.001.

In the QS questionnaire administered to IAC instructors to survey their satisfaction with participation in the multisport training course, question 9 (related to the perception of children’s motor skills improvement), resulted in the lower. The Friedman analysis confirmed that the items of the questionnaire of satisfaction significantly scored differently (*p* < 0.001). However, the post hoc highlighted that only question 9 significantly differed from several other questions, as reported in Figure 3.

The instructors’ self-reporting of the teaching styles surveyed by the TSQ was implemented by observing and analyzing some lessons by the IFITS (Table 4). The analysis showed a significant difference in pre-intervention practice teaching styles (*p* = 0.038), while in post-intervention, significant differences were detected in command (*p* = 0.01), practice (*p* < 0.001), inclusion (*p* < 0.001), guided discovery (*p* = 0.010), management (*p* = 0.03), and production (*p* = 0.010).

The physical commitment of the children depending on how the instructors conducted the lessons, as reported by the SOFIT analysis (Table 5), was similar in Ch-IAC and Ch-CON, confirming that there was no difference between IAC and I-CON conduction before IAC training. Conversely, after the training, IAC instructors improved their conduction: compared to I-CON, children belonging to IAC teams spent less time standing (*p* = 0.043), benefited from lessons having less management (*p* < 0.001), reduced fitness (*p* = 0.023), and more game (*p* = 0.017) contents, and received more lessons outside topics (*p* < 0.001).

**Figure 4:**
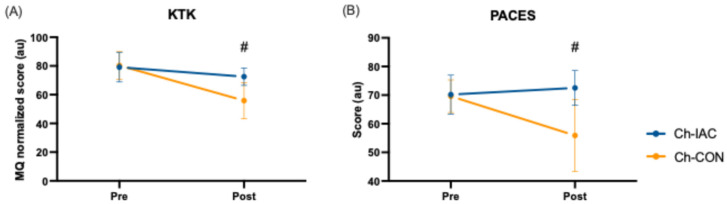
Time × group interactions in children’s motor skills and enjoyment of physical activity. (**A**) KTK (Körperkoordinationtest für Kinder) motor quotient; (**B**) PACES (Physical Activity Enjoyment Scale) total score. MQ = motor quotient; Ch-IAC = children from teams of instructors attending the training course; Ch-CON = children of the instructors’ control group. # = significant time × group interaction (*p* < 0.001).

The children’s testing sessions showed that Ch-IAC (i.e., the children of the teams of the instructors who attended the training course) outperformed Ch-CON in several parameters. In coordination skills, a significant interaction (time × group) was retrieved in the normalized motor quotient of the KTK (F = 23.549, *p* < 0.001, ηp^2^ = 0.056), indicating that Ch-IAC had a higher normalized motor quotient than Ch-CON in the post-test (Figure 4, panel A). Similarly, Ch-IAC also enjoyed the activity more than Ch-CON: a significant interaction (time × group) of PACES questionnaire total score (F = 94.463, *p* < 0.001, ηp^2^ = 0.193) was retrieved (Figure 4, panel B).

**Figure 5:**
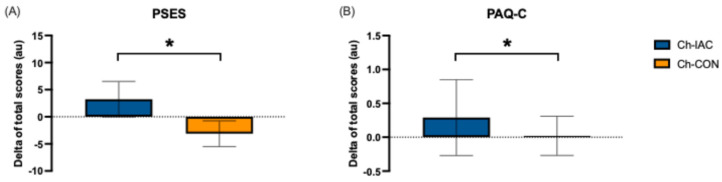
Children’s self-efficacy and physical activity level variations (delta = post–pre values). (**A**) PSES (physical self-efficacy scale for children) delta scores; (**B**) PAQ-C (Physical Activity Questionnaire for Older Children) delta scores. Ch-IAC = children from teams of instructors attending the training course; Ch-CON = children of instructors’ control group. * = *p* < 0.001.

Instructors’ participation in the training course considerably affected children’s self-efficacy and physical activity levels. The analysis of delta values (post–pre) of PSES (Figure 5, panel A) showed that Ch-IAC positively increased self-efficacy compared to Ch-CON, which lowered (*p* < 0.001). Similarly, PAQ-C delta analysis (Figure 5, panel B) showed higher positive changes in Ch-IAC physical activity levels than Ch-CON (*p* < 0.001).

**Figure 6:**
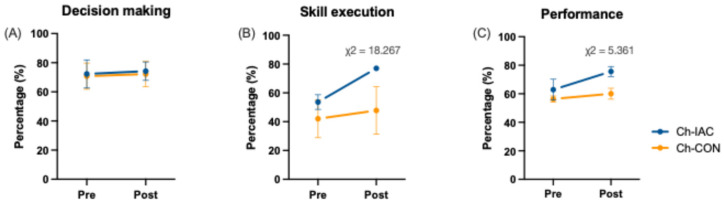
Match analysis results by the game performance assessment instrument (GPAI) related to decision making (**A**), skill execution (**B**), and performance (**C**). Significant differences by the Chi-squared analysis are reported.

The match analysis through the GPAI instrument (Figure 6) showed an increase in Ch-IAC efficient skill execution and, consequently, in general match performance. No changes were detected between pre- and post-intervention decision-making.

### Mediation and Moderation Analysis

**Figure 7:**
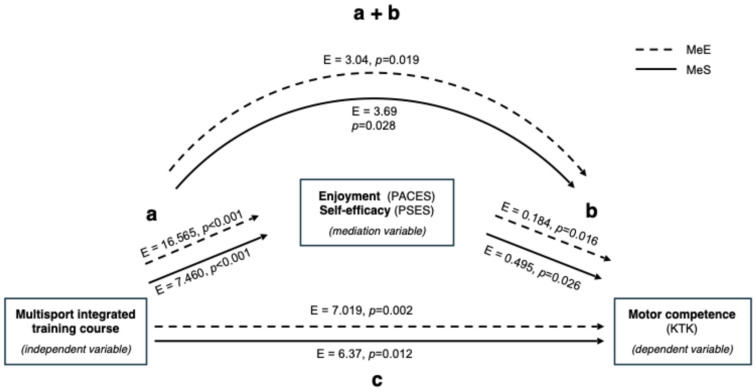
Flow chart of the mediation analysis. MeE = enjoyment mediation; MeS = self-efficacy mediation. a = effect of the independent on the mediation variable; b = effect of the mediation on the dependent variable; c = effect of the independent on the dependent variable (direct effect); a + b = effect of the independent on the dependent variable mediated by the mediation variable (indirect effect).

Figure 7 depicts the effects of enjoyment and self-efficacy as mediators in the relationship between independent (multisport-integrated training course) and dependent (motor competence) variables. The multisport-integrated training course had a significant effect on motor competence (c: E = 7.019, *p* = 0.002) and enjoyment (a: E = 16.565, *p* < 0.001). A significant effect of enjoyment on motor competence was found (b: E = 0.184, *p* = 0.016). The MeE, revealed that the indirect effect (a + b) of enjoyment is significant (a + b: E = 3.04, *p* < 0.0019), so the enjoyment mediates the effect of the intervention on motor competence. In the MeE, a significant total effect was detected (E = 10.06; *p* < 0.001). As in MeE, the direct effect (even if significant) resulted lower than the total effect; it is possible to notice that enjoyment partially mediates the effect of the intervention on motor competence.

The self-efficacy mediation analysis (MeS) revealed that intervention had a significant effect on motor competence (c: E = 6.37, *p* = 0.012) and Self-efficacy (a: E = 7.460, *p* < 0.001). A significant effect of self-efficacy on motor competence was found (b: E = 0.495, *p* = 0.026). The MeS, revealed that the indirect effect (a + b) of self-efficacy is significant (a + b: E = 3.69, *p* < 0.0028), so the self-efficacy mediates the effect of the intervention on motor competence. In the MeS, a significant total effect was detected (E = 10.06; *p* <0.001). As in MeS, the direct effect (even if significant) resulted lower than the total effect; similarly to MeE, it is possible to notice that self-efficacy partially mediates the effect of the intervention on motor competence.

The PAQ-C mediation analysis (MeP) revealed that intervention had a significant effect on motor competence (c: E = 9.66, *p* < 0.001) and PAQ-C (a: E = 0.317, *p* < 0.001). No significant effect of PAQ-C on motor competence was found (b: E = 1.28, *p* = 0.553). In the MeP, a significant total effect was detected (E = 10.06; *p* <0.001). The MeP, revealed that the indirect effect (a + b) of PAQ-C is not significant (a + b: E = 0.405, *p* = 0.556), so the amount of physical activity does not mediate the effect of the intervention on motor competence.

**Figure 8:**
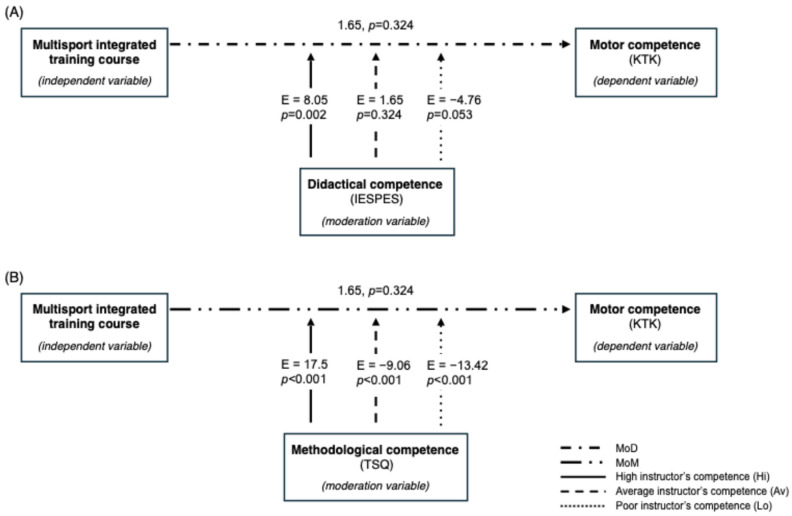
Flow chart of the moderation analysis of the role of didactical competence (**A**) and methodological competence (**B**). MoD = didactical competence moderation; MoM = methodological competence moderation. In (**A**), the moderation analysis of the role of didactical competence (MoD) is shown., the full line refers to a high instructors’ competence (Hi), the double-dashed line to an average instructor’s (Av) competence and the dotted line to a poor instructor’s competence (Lo).

The moderation analysis (Figure 8) of didactical competencies (MoD) showed that a single multisport training course alone could not fully explain the DV results (E = 1.65, *p* = 0.324). Indeed, high didactics competence of instructors (Hi) positively influences the motor competence in children (E = 8.05, *p* = 0.002), an average instructor’s didactics competence (Av) brought a little but not significant improvement in motor competence (E = 1.65, *p* = 0.324), and a poor or just sufficient instructors’ competence in didactics (Lo) might lead to an aggravation of motor competence (Lo: E = −4.76, *p* = 0.053; Av: E = −9.06, *p* < 0.001). Furthermore, the MoD interaction’s effect shows that, for every increase in units in methodological competence, the effect of the intervention becomes more positive of 11.11 units (E = 11.11, *p* < 0.001).

About MoM, the higher the instructor competence (Hi), the higher the motor competence (E = 17.5, *p* = 0.001). Moreover, the MoM interaction’s effect shows that, for every increase in units in methodological competence, the effect of the intervention becomes more positive of 5.94 units (E = 5.94, *p* = 0.057). Hence, as the number of teaching styles used increases, it partially moderates the effect of intervention.

## 4. Discussion

The primary aim of this study was to investigate the effects of multisport-integrated model training, which a sample of rugby instructors (IAC) participated in. Thanks to training, IAC instructors significantly improved their didactic and methodological competencies, as evaluated through the internship evaluation sheet in physical education and sports (IESPES), the Instrument for Identifying the Teaching Style (IFITS), and the self-report referring to the Teaching Style Questionnaire (TSQ).

**Figure 9:**
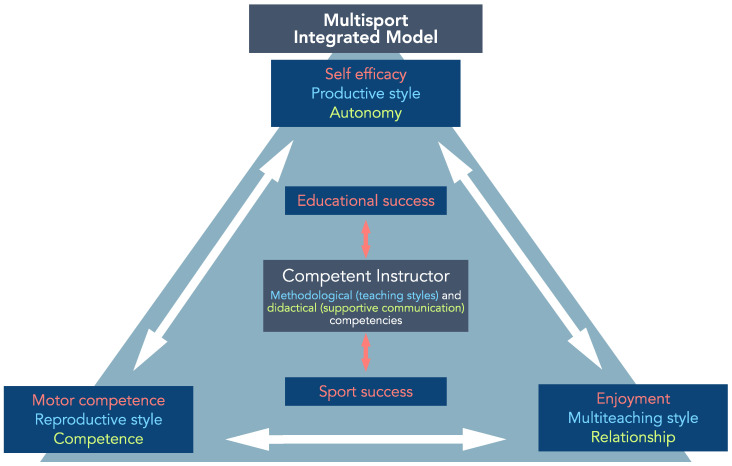
The framework of the integrative multisport model: educational and sports success is strictly related to the key elements characterizing the need supportive communication integrated with the utilization of different teaching styles (autonomy, competence, and relationship). Both instructors and children are related to these key elements.

In the integrative multisport model we presented, educational and sports success are strictly related and depend on instructors’ competence. Figure 9 graphically depicts the multisport-integrated model and how educational and sports success are considered part of the same system. In educational and sports success, autonomy, competence, and relationship are the keys to supportive communication based on a pupil-centered approach. Teaching styles are related to autonomy (production styles centered on pupil), competence (reproduction styles centered on motor competencies), and relationship (multi-teaching based both on education in sport and through sport with adaptation to the needs of children). Autonomy, general competence, and relationships help build the children’s psychological aspects (such as self-efficacy and enjoyment) and motor competence. A competent instructor based on methodological competence (teaching styles) and didactics competence (elements aimed to need supportive communication: communication capacity, motivation capacity, and didactic organization) is the core of this integrated multisport system.

Based on the results of the IESPES instrument evaluating the internship’s success, IAC improved total score, communication skills, didactics organization, motivation, and personal competence. These findings agree with Kirkpatrick [22], who found that proposing an adequate training course, alternating theoretical topics, debates, and internships with instructors and children could enhance instructors’ didactic and methodological competence.

The self-reported teaching styles questionnaire showed that the IAC instructors used many more different teaching styles at the end of the multisport training course. Despite the frequent use of reproduction styles (70.9%), thanks to the training course, they learned to use production styles, such as those suggested by Da Silva, for team-oriented sports because helping children develop decision-making and playing skills [39,40,41]. Instructors improved their overall methodological competence by learning to manage the integration of reproduction and production teaching styles with the multi-teaching approach.

The use of an integrated multisport approach combined with methodological and didactical competence (as we considered through the statistical analysis of moderators) could improve socio-psychological aspects and self-awareness in children because it helps them develop autonomy, motor competence, and relationships with the pairs and adults [14,42,43].

The SOFIT’s results about the conduction of physical activity performed by the pupils trained by IAC found a significant reduction in the children’s time standing still (from 33.6% to 11.2% of the total lesson time) spent in the instructors’ management (from 7.8% to 2.6%) and fitness (from 13.6% to 2.1%), and a significant increase in instructors’ promotion of outside physical activity (from 0% to 4.4%). The decrease in the time spent standing still and in management presumably highlights that children were more involved in physical activity and, therefore, that IAC instructors found other strategies to manage the children, such as sitting during explanations (as the increase in time spent sitting evidence). Indeed, the sitting posture allows for a minor possibility of uncontrolled movement of pupils, generating less problematic and dispersive situations during group management, with fewer organizational difficulties, as the reduction in management time retrieved by both SOFIT (−5.1%) and IFITS (−19.7%) analysis confirms. The decrease in the time spent on fitness (−11.6%) can be compensated by an increase in time spent on games (19.9%). According to Feigenbaum [44], children of this age should become fit and conditioned through different proposals, not only aimed at mere physical fitness but also through games that are more motivating proposals than mere exercises that make them highly involved and adequately conditioned. General conditioning by games could result in a lower perception of fatigue and higher motor control. These data, according to GPAI outcomes for execution skills and game performance, showed a possible improvement in IAC after the multisport training course because the more fitness (acquired by general games), the more precision in specific skills and effective performance. The integration of motor control in conditioning through games is the most effective way to reach a positive effect in situational sports performance due to an immediate transfer. Instructors also increased the promotion of outside activity (which was highlighted by the previous analysis with SOFIT) thanks to the multisport training course, as the increment of PAQ-C values of Ch-IAC confirms. These results demonstrate how a multisport-integrated approach contributes to the development of healthy lifestyles and public health and, therefore, to educational success based on physical literacy [45].

When addressing the effects of the multisport training of IAC on children’s outcomes, this study’s results evidenced significant differences in motor competence and game performance in favor of Ch-IAC compared to Ch-CON. The multisport-integrated approach positively influenced the motor coordination of children taught by IAC instructors, possibly because they proposed different types of activities and stimuli than I-CON, making the children more coordinated and motor competent. According to the literature, children who are stimulated to try different kinds of basic sports skills and activities are better supported in continuing motor and sports practice as they overcome the proficiency barrier [46,47,48]. As previously highlighted, considering the PAQ-C results, the multisport-integrated approach can increase the children’s amount of physical activity as instructors increase the promotion of sports practice and the subsequent children’s knowledge and the chance to choose additional sports activities in their free time [49,50]. Children who are not stimulated to vary motor skills and sports activities might not be able to acquire sufficient fitness at later ages. Therefore, the results of the present study represent a valuable indicator of how such an approach based on multisport-integrated practice can positively impact the well-being and health of the children’s future [51,52,53].

The positive outcomes of PACES of Ch-IAC show that children enjoyed the multisport activities proposed and were involved in activities with different proposals that suit them best. The increase in PSES score Ch-IAC also demonstrates that the possibility to try different sports and learn different skills enhanced children’s self-efficacy [18,54,55,56]. Furthermore, given the positive outcomes from the instructors’ questionnaire of satisfaction analysis, this educational training program results in a solid platform for sports and educational success. Considering this, instructors and sports societies should align their interventions with these findings [22].

The mediation and moderation analysis further investigated the effectiveness and transferability of the multisport-integrated training course on children. The results emerging from the mediation analysis showed that enjoyment and self-efficacy partially mediate the effect of the multisport intervention on motor competence, so they partially could amplify the effects on motor competence in children [18,54]. The moderation analysis showed that different levels of didactical and methodological competence can improve or aggravate motor competence in children. The more the instructors’ didactical and methodological competence, the more the children’s motor competence; the less didactical and methodological competence the instructors have, the less the children’s motor competence. For this purpose, the use of a need supportive communication integrated with motivational, personal, and organizational competence (didactical approach) and a multi-teaching approach (methodological competence) might improve the children’s motor competence [14,42,50].

Using a multi-teaching style and active reflection approach, stimulated above all by greater use of productive styles based on question-delving into the “why” and “how” of human movement, is a way to encourage non-linear pedagogies. This way, it has been possible to administer a varied and non-linear approach by modifying task, environmental, or organismic constraints, therefore not addressing what to do (as often authors claim in literature) but how to do it (increasing a diversified teaching styles approach), which ended in more significant effects on learning in the experimental group (Ch-IAC) compared to the control group [50]. The nonlinearity of learning processes must be interpreted holistically, considering not only the individual and the effects that a proposal could determine on the different personal areas but also the group in which the individual is inserted and the context that could determine the necessity of finding new ways to offering sports experiences in relation to the children’s needs [57]. Based on nonlinear pedagogy, a multisport approach stimulates variants integrated with the methodological ones related to teaching styles.

The literature evidenced how the acritical use of sports activities, caused by the implicit selectivity of the sport, is incompatible with an active long lifestyle [58,59]. Also, a multisport interpretation, as a summary of different sports disciplines, not mediated by reflective practice and not contextualized, could lead to an excess of competition, which results in one of the most common causes of dropouts in youth [60,61]. Young athletes must be stimulated to reflect on their limits or competence, act reflectively, and self-check their practice through questions to avoid and prevent this problem.

Further consideration arises concerning the relationship between a nonlinear approach based on methodological competence (multi-teaching style practice) and the instructors’ didactic competence related to supportive communication and organization of contents competence [62]. The notable relationship that emerged demonstrates how methodological and didactic competence are strictly related. The instructors’ skills in varying teaching and sports approaches further let them acquire a better competence in supportive communication, organizational aspects of didactics, and lesson management. Using a nonlinear pedagogy by varying proposals through multisport and multi-teaching approaches and reflective practice (as presented in the training course that has been the object of the present study) can stimulate pupil-centered teaching with the aim to generate a children’s task-oriented motivational climate essential to acquire adequate motor skills [50,63,64]. Furthermore, a multi-teaching approach might favor the use of need-supportive communication and didactics organization through educational experiences that satisfy children’s psychological needs (autonomy, competence, relationship) necessary to lead to sports and educational success [14].

Regarding Vigotskij’s zones of proximal development [65], adapting teaching styles and communication methods to find the proper stimulation through a holistic perspective does not mean forgetting the technical aspects of the discipline but intentionally inserting them in the didactic-methodological approach to guarantee a more efficient acquisition.

Educational didactics in sports, long-lived and sustainable, do not focus on performance but must apply the different levels of motor competence through methodological and didactic strategies to embrace children’s rights [66,67] and guarantee continuity of practice at group and sports club levels [68,69]. Long-life sports practice is essential and should include inclusive didactics oriented to a progressive consciousness related to one’s limits of competence and possible improvements, representing a concrete pillar of youth life and physical activity habits [53]. From this perspective, to move from an interpretation of sports disciplines as an objective to an interpretation aimed at educational purposes, training instructors’ courses aimed at modifying the mentality, approaches, methods and teaching methods with which sports practice is traditionally proposed at a youth level becomes indispensable. The success of a youth instructor should not be evaluated through the performance obtained by children but in avoiding dropouts and promoting future continuity of individual practice by stimulating motivation to play sports in the current discipline or others.

**Figure 10:**
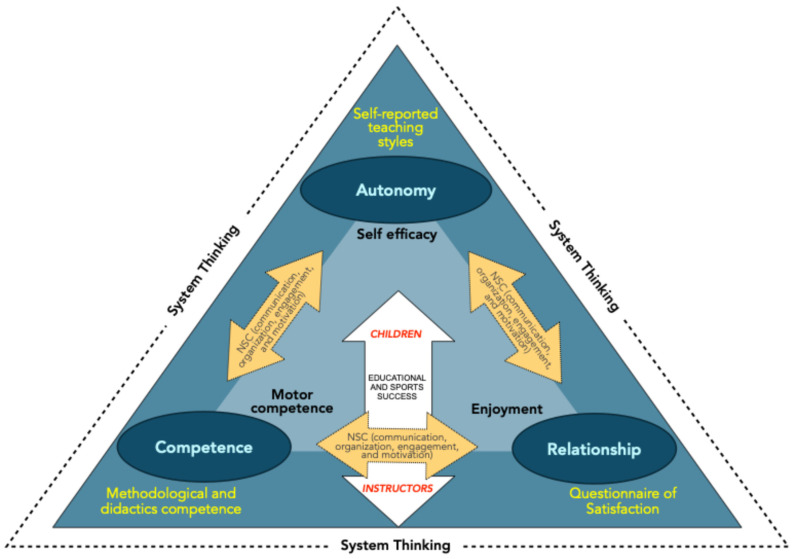
Conceptual model of the instructor-children system of the multisport-integrated training approach and its investigation. The two triangles represent the instructors’ (blue) and children’s (light blue) systems. The fundamental topics of the need for supportive communication are shown at the vertexes. For every dimension (autonomy, competence, and relationship), this study assessed instructors’ perception of the teaching styles used, methodological and didactic competence, and appreciation for the training course (yellow text). For the same dimensions, the children’s self-efficacy, motor competence, and enjoyment were measured (black text). The children-instructor relationship is the basis of educational and sports success. NSC = need supportive communication.

Instructors’ and children’s systems are on the same plan and converge into a unique model, according to the system thinking approach (Figure 10), in which leverage, and resistance points can be individuated. Leverage points for both instructors and children are autonomy (self-reported teaching styles used and self-efficacy, respectively), competence (methodological and didactics competence, and motor competence, respectively), and relationship (questionnaire of satisfaction and enjoyment, respectively) [70]. Resistance points can be the relationship between instructors and children and the actual vision of several Italian sports societies, which consider sports and educational success as detached systems, with the primary importance addressed to performance [70]. This model integrates instructors’ and children’s plans, builds a relationship based on a need for supportive communication and the vision of sports and educational success as attached parts and fundamental bases for constructing instructors and athletes of every category from childhood to adulthood.

### Limits of This Study

In this study, the multisport model was limited to instructors interacting in a youth sports contest, not accounting for relationships with high-performance or amateur sports aimed at the well-being promotion of later ages. The model did not fully account for the possible conflicts that could emerge in the actual situation by converging the subsystems of competitive sport (ego-oriented, selective, and often considered primary in Italian reality) and educational sport (task-oriented based on an inclusion system). The conflicts between the two contrasting situations could generate an imbalance challenging to overcome in sports clubs [71].

The model also concerns the specific reality of rugby that cannot be generalized in other contests and sports disciplines. Based on the outcomes of this initial study, we can assert that the project’s structure has the potential to shape our understanding of sports and sports associations in formative and educational terms. Notably, a multisport activity has been found to significantly enhance the children’s motor competence and play capacity, leading to higher sporting success.

## 5. Conclusions

The rugby instructors attending a multisport-integrated training course considerably improved their didactic and methodologic competencies in conducting the sports activity with their pupils. The success of the multisport model (Figure 10) can be ascribed to having integrated the children’s and instructors’ domains related to the autonomy, competence, and relationship dimensions according to supportive communication, which, under the system thinking perspective, considers interconnections among setting, activity, and social dynamics variables of the minirugby environment. Participation, individual competence and performance of both instructors and minirugby players were favored by the multisport-integrated training.

The future development of this type of research is represented by longitudinal studies extended to different sports disciplines and social realities, which can, in the long term, collect data/observations for comparisons between different environmental and cultural contexts.

## Figures and Tables

**Table 1:** Participants’ demographics and grouping specifics.

Participants	Variable	Experimental (IAC, Ch-IAC)	Control (CON, Ch-CON)
Instructors	N	25 (21 m, 4 f)	25 (24 m, 1 f)
	Age (y)	36.2 ± 14.4	40.0 ± 12.8
	Teaching experience (y)	5.36 ± 6.0	7.0 ± 5.0
Children	N	109 (106 m, 3 f)	111 (110 m, 1 f)
	Age (y)	9.6 ± 1.1	9.6 ± 1.0
	Weight (kg)	36.5 ± 8.3	35.4± 6.5
	Height (m)	1.38 ± 0.07	1.39 ± 0.07
	BMI (kg/m^−2^)	19.1 ± 3.6	18.2 ± 2.8

**Table 2:** Instructors’ didactic approach from the internship evaluation sheet in physical education and sports (IESPES) questionnaire.

IESPES Parameters	Time Point	IAC	I-CON
Communication	Pre	2.9 ± 0.7	3.1 ± 0.5
	Post	3.7 ± 0.4	3.0 ± 0.5
	∆ (post–pre)	0.8 ± 0.4 §	0.0 ± 0.3
Didactics organization	Pre	2.9 ± 0.7	3.0 ± 0.8
	Post	3.7 ± 0.7	3.2 ± 0.6
	∆ (post–pre)	0.8 ± 0.6 §	0.2 ± 0.5
Motivation and personal competence	Pre	2.4 ± 0.7	2.6 ± 0.7
	Post	3.6 ± 0.4	2.6 ± 0.7
	∆ (post–pre)	1.1 ± 0.6 §	0.1 ± 0.5
Total mean score	Pre	2.8 ± 0.5	2.9 ± 0.3
	Post	3.7 ± 0.3	3.0 ± 0.4
	∆ (post–pre)	0.9 ± 0.3 §	0.1 ± 0.2

Values (mean ± SD) are expressed in AU (score). IAC = instructors attending the training course; I-CON = control. Significant values: § = time × group interaction (*p* < 0.05).

**Table 3:** Instructors’ self-reported teaching styles from the teaching styles questionnaire (TSQ).

Teaching Style	Time Point	IAC	I-CON
Multi-teaching	Pre	3.5 ± 1.2 *	2.9 ± 0.8
	Post	4.8 ± 1.3 *	2.7 ± 0.8
	∆ (post–pre)	1.3 ± 0.9 *	−0.2 ± 0.5
Reproduction	Pre	2.6 ± 0.6 *	2.2 ± 0.5
	Post	3.0 ± 0.8 *	2.2 ± 0.5
	∆ (post–pre)	0.4 ± 0.5 *	0.0 ± 0.0
Production	Pre	0.9 ± 0.9	0.6 ± 0.8
	Post	1.8 ± 1.0 *	0.4 ± 0.7
	∆ (post–pre)	0.9 ± 0.8 *	−0.2 ± 0.5

Values (mean ± SD) are expressed in N (number of reported occurrences). IAC = instructors attending the training course; I-CON = control. Significant values: * = different than I-CON (*p* < 0.05).

**Table 4:** Percentages of teaching styles used by the instructors as assessed by the Instrument for Identifying the Teaching Style (IFITS).

Teaching Style	Time Point	IAC	I-CON
Command	Pre	23.3 ± 21.9	4.9 ± 8.8
	Post	39.4 ± 13.6 *	8.8 ± 15.8
	∆ (post–pre)	16.0 ± 27.6	3.9 ± 13.2
Practice	Pre	37.6 ± 28.0 *	62.2 ± 24.6
	Post	20.0 ± 10.0 *	59.5 ± 21.2
	∆ (post–pre)	−17.0 ± 32.7	−2.7 ± 13.4
Reciprocal	Pre	N/U	N/U
	Post	3.5 ± 6.9	N/U
	∆ (post–pre)	3.5 ± 6.9	N/A
Self-check	Pre	N/U	N/U
	Post	N/U	N/U
	∆ (post–pre)	N/A	N/A
Inclusion	Pre	N/U	N/U
	Post	8.2 ± 1.8 *	N/U
	∆ (post–pre)	8.2 ± 1.8 *	N/A
Guided discovery	Pre	N/U	N/U
	Post	10.5 ± 9.3 *	N/U
	∆ (post–pre)	10.5 ± 9.3 *	N/A
Convergent discovery	Pre	N/U	N/U
	Post	N/U	N/U
	∆ (post–pre)	N/A	N/A
Going beyond	Pre	N/U	N/U
	Post	N/U	N/U
	∆ (post–pre)	N/A	N/A
Management	Pre	39.1 ± 11.3	32.9 ± 21.7
	Post	19.4 ± 10.4 *	31.7 ± 7.9
	∆ (post–pre)	−19.7 ± 17.3 *	−1.2 ± 17.3
Total Reproduction	Pre	60.9 ± 11.3	67.1 ± 21.7
	Post	70.9 ± 8.3	68.3 ± 7.9
	∆ (post–pre)	10.1 ± 15.7	1.2 ± 17.3
Total Production	Pre	N/U	N/U
	Post	10.5 ± 9.3 *	N/U
	∆ (post–pre)	10.5 ± 9.3 *	N/A

Values (mean ± SD) are expressed in percentage of the lesson total time (%). IAC = instructors attending the training course; I-CON = control. N/U = not used. N/A = not applicable. Significant values: * = different than I-CON (*p* < 0.05).

**Table 5:** Children’s physical commitment during the lessons due to the instructors’ conduction, as from the System for Observing Fitness Instruction Time (SOFIT) analysis.

Children’s Commitment		Time Point	IAC	I-CON
Children’s activity	Lay	Pre	1.2 ± 2.6	0.3 ± 0.9
		Post	0.9 ± 1.4	0.4 ± 0.8
		∆ (post–pre)	−0.3 ± 3.3	0.1 ± 1.3
	Seated	Pre	3.1 ± 4.6	2.5 ± 3.6
		Post	4.3 ± 3.3	3.7 ± 3.0
		∆ (post–pre)	1.2 ± 4.4	1.2 ± 4.5
	Stand	Pre	33.6 ± 16.3	26.6 ± 14.4
		Post	11.2 ± 5.6 *	20.2 ± 12.3
		∆ (post–pre)	−22.4 ± 20.4	−6.4 ± 22.9
	Moderate	Pre	25.1 ± 11.9	28.5 ± 11.6
		Post	34.8 ± 9.0	31.2 ± 11.1
		∆ (post–pre)	9.7 ± 15.1	2.8 ± 18.2
	Vigorous	Pre	37.0 ± 14.1	43.2 ± 12.2
		Post	48.8 ± 10.2	44.4 ± 12.8
		∆ (post–pre)	11.9 ± 21.0	1.3 ± 18.7
Lesson content	Management	Pre	7.8 ± 3.6	6.4 ± 2.7
		Post	2.6 ± 1.0 *	7.5 ± 1.8
		∆ (post–pre)	−5.1 ± 3.3 *	1.1 ± 3.1
	Knowledge	Pre	21.6 ± 9.0	21.2 ± 11.0
		Post	23.0 ± 7.1	17.9 ± 17.4
		∆ (post–pre)	1.4 ± 12.8	−3.3 ± 20.9
	Fitness	Pre	13.6 ± 19.2	19.7 ± 21.0
		Post	2.1 ± 4.0 *	12.1 ± 9.4
		∆ (post–pre)	−11.6 ± 18.0	−7.6 ± 18.8
	Skills	Pre	44.7 ± 26.2	39.7 ± 28.4
		Post	40.5 ± 12.5	31.4 ± 20.9
		∆ (post–pre)	−4.2 ± 28.6	−8.3 ± 30.1
	Game	Pre	11.9 ± 15.7	13.0 ± 17.9
		Post	31.8 ± 11.4 *	31.2 ± 32.9
		∆ (post–pre)	19.9 ± 16.5	18.2 ± 30.1
Coaches’ interaction	Inside lesson topics	Pre	38.5 ± 12.5	33.0 ± 12.4
		Post	42.1 ± 11.8	38.0 ± 13.4
		∆ (post–pre)	3.6 ± 11.4	5.1 ± 9.4
	Outside lesson topics	Pre	0.0 ± 0.0	0.0 ± 0.0
		Post	4.4 ± 3.2 *	0.0 ± 0.0
		∆ (post–pre)	4.4 ± 3.2	0.0 ± 0.0
	No interaction	Pre	61.3 ± 12.2	67.0 ± 12.4
		Post	54.6 ± 14.5	62.1 ± 13.3
		∆ (post–pre)	−6.8 ± 13.0	−5.0 ± 9.3

Values (mean ± SD) are expressed in percentage of the lesson total time (%). IAC = instructors attending the training course; I-CON = control. Significant values: * = different than I-CON (*p* < 0.05).

## Data Availability

The data supporting the main findings of this study are available on reasonable request with access granted to researchers meeting the criteria for access to confidential data. The data repository is Zenodo, at https://doi.org/10.5281/zenodo.13999101 (URL accessed on 27 October 2024).

## Appendix A. IESPES Items’ Descriptors

**Table:**

Items	Descriptors ^1^
Verbal communication	Comprehension; logic; choice of words of appreciation; choice of words of reproach; use of metaphors/narration.
Voice and paralanguage	Volume; Timbre; Modulation; expository rhythm; use of silences and pauses.
Non-verbal communication	Facial expression; eye gaze; eye contact; posture; proxemics gestures.
Specific didactics	Choice of exercises; progression; demonstration; correction; adaptation of the proposal.
Elements of organizational references	Location of tools; location in space of learners; safety/prevention/assistance rules; use of conceptual diagrams/charts/illustrations; time management (activity times; question times; organizational times).
Psychological references	Interest aroused; emulation level; expectations/requests; reinforcement; contextualization.
Personal competence	Charisma; accuracy; confidence; empathy; self-monitoring

^1^ Instructors were evaluated on the five descriptors for each item. The descriptors were scored as 0 (=inadequate) or 1 (=adequate) point, returning a score ranging from 0 and 5 for each item. The final score was calculated as the mean of all item’s scores.
